# Ambroxol improves lysosomal biochemistry in glucocerebrosidase mutation-linked Parkinson disease cells

**DOI:** 10.1093/brain/awu020

**Published:** 2014-02-25

**Authors:** Alisdair McNeill, Joana Magalhaes, Chengguo Shen, Kai-Yin Chau, Derralyn Hughes, Atul Mehta, Tom Foltynie, J. Mark Cooper, Andrey Y. Abramov, Matthew Gegg, Anthony H.V. Schapira

**Affiliations:** 1 Department of Clinical Neurosciences, Institute of Neurology, University College London, UK; 2 Bioinformatics Unit, Source Bioscience, Nottingham, UK; 3 Lysosomal storage disorders unit, Royal Free Hospital, London, UK; 4 Sobell Department of Motor Neuroscience and Movement Disorders, Institute of Neurology, University College London, UK; 5 Department of Molecular Neuroscience, Institute of Neurology, University College London, UK

**Keywords:** Parkinson’s disease, ambroxol, lysosome, Gaucher disease, glucocerebrosidase

## Abstract

Gaucher disease is caused by mutations in the glucocerebrosidase gene, which encodes the lysosomal hydrolase glucosylceramidase. Patients with Gaucher disease and heterozygous glucocerebrosidase mutation carriers are at increased risk of developing Parkinson’s disease. Indeed, glucocerebrosidase mutations are the most frequent risk factor for Parkinson’s disease in the general population. Therefore there is an urgent need to understand the mechanisms by which glucocerebrosidase mutations predispose to neurodegeneration to facilitate development of novel treatments. To study this we generated fibroblast lines from skin biopsies of five patients with Gaucher disease and six heterozygous glucocerebrosidase mutation carriers with and without Parkinson’s disease. Glucosylceramidase protein and enzyme activity levels were assayed. Oxidative stress was assayed by single cell imaging of dihydroethidium. Glucosylceramidase enzyme activity was significantly reduced in fibroblasts from patients with Gaucher disease (median 5% of controls, *P = *0.0001) and heterozygous mutation carriers with (median 59% of controls, *P = *0.001) and without (56% of controls, *P = *0.001) Parkinson’s disease compared with controls. Glucosylceramidase protein levels, assessed by western blot, were significantly reduced in fibroblasts from Gaucher disease (median glucosylceramidase levels 42% of control, *P < *0.001) and heterozygous mutation carriers with (median 59% of control, *P < *0.001) and without (median 68% of control, *P < *0.001) Parkinson’s disease. Single cell imaging of dihydroethidium demonstrated increased production of cytosolic reactive oxygen species in fibroblasts from patients with Gaucher disease (dihydroethidium oxidation rate increased by a median of 62% compared to controls, *P < *0.001) and heterozygous mutation carriers with (dihydroethidium oxidation rate increased by a median of 68% compared with controls, *P < *0.001) and without (dihydroethidium oxidation rate increased by a median of 70% compared with controls, *P < *0.001) Parkinson’s disease. We hypothesized that treatment with the molecular chaperone ambroxol hydrochloride would improve these biochemical abnormalities. Treatment with ambroxol hydrochloride increased glucosylceramidase activity in fibroblasts from healthy controls, Gaucher disease and heterozygous glucocerebrosidase mutation carriers with and without Parkinson’s disease. This was associated with a significant reduction in dihydroethidium oxidation rate of ∼50% (*P < *0.05) in fibroblasts from controls, Gaucher disease and heterozygous mutation carriers with and without Parkinson’s disease. In conclusion, glucocerebrosidase mutations are associated with reductions in glucosylceramidase activity and evidence of oxidative stress. Ambroxol treatment significantly increases glucosylceramidase activity and reduces markers of oxidative stress in cells bearing glucocerebrosidase mutations. We propose that ambroxol hydrochloride should be further investigated as a potential treatment for Parkinson’s disease.

## Abstract (precis)

Heterozygous *GBA* gene mutations are the most frequent Parkinson’s disease risk factor. Using Parkinson’s disease patient derived fibroblasts McNeill *et al*. show that heterozygous *GBA* mutations reduce glucosylceramidase activity, and are associated with endoplasmic reticulum and oxidative stress. Ambroxol treatment improved glucosylceramidase activity and reduced oxidative stress in these cells.

## Introduction

The autophagy–lysosome system plays a key role in degrading the misfolded proteins that form the abnormal protein accumulations that occur in the common late onset neurodegenerative diseases. In concert with the proteasome, the autophagy–lysosome system degrades tau, the proteins that form neurofibrillary tangles in Alzheimer’s disease (Lee *et al.*, 2013). Markers of dysfunctional autophagy have been described in motor neuron disease spinal cord (Otomo *et al.*, 2012), and the autophagy–lysosome system plays a role in degrading superoxide dismutase 1 and TBP43 (now known as TARDBP) (Otomo *et al.*, 2012). There is also strong evidence for a role of this system in the aetiopathogenesis of Parkinson’s disease (Goker-Alpan, *et al.*, 2010; Ebrahimi-Fakhari *et al.*, 2012; Houlden *et al.*, 2012). Accumulation of p62 and LC3-II, markers of dysfunction of the autophagy–lysosome system, has been described in post-mortem Parkinson’s disease brain (Alvarez-Erviti *et al.*, 2010; Dehay *et al.*, 2013). In addition cell biology studies have demonstrated that inhibition of the autophagy–lysosome system, and in particular chaperone-mediated autophagy, is associated with elevation of alpha-synuclein protein levels (Alvarez-Erviti *et al.*, 2010). Recently, mutations in the glucocerebrosidase gene (*GBA*), which encodes the lysosomal hydrolase glucosylceramidase deficient in Gaucher disease, have been identified as a risk factor for the development of Parkinson’s disease (Sidransky *et al.*, 2009; Sidransky and Lopez, 2012), dementia with Lewy bodies (Nalls *et al.*, 2013) and a subtype of Alzheimer’s disease (Tsuang *et al.*, 2012).

Three broad clinical subtypes of Gaucher disease are recognized (Balwani *et al.*, 2010). In type I (non-neuronopathic) Gaucher disease, patients develop combinations of blood dyscrasia, hepato-splenomegaly and bone disease (Balwani *et al.*, 2010). Patients with types II (acute neuronopathic) and III (chronic neuronopathic) Gaucher disease present with a predominantly neurodegenerative syndrome in childhood or early adulthood (Tajima *et al.*, 2009). Several hundred *GBA* mutations have been reported, but the most common are the N370S and L444P missense mutations (Hruska *et al.*, 2008). There is no precise genotype–phenotype correlation but patients with the N370S allele generally develop non-neuropathic disease (Balwani *et al.*, 2010). The clinical manifestations of Gaucher disease are associated with lysosomal accumulation of the glucosylceramidase substrates glucosylceramide and glucosylsphingosine (Balwani *et al.*, 2010). Patients with Gaucher disease have an 8–12% chance of developing Parkinson’s disease by age 80 (Rosenbloom *et al.*, 2011) whereas the risk in heterozygous *GBA* mutations carriers is 10–15% by age 80 (McNeill *et al.*, 2012).

The mechanisms by which *GBA* mutations predispose to neurodegeneration remain unclear. However, post-mortem studies have demonstrated severe loss of glucosylceramidase enzyme activity in brain tissue from patients with Gaucher disease and in the substantia nigra of patients with Parkinson’s disease (Mazzulli *et al.*, 2011; Gegg *et al.*, 2012) and patients with Lewy body dementia (Kurzawa-Akanbi *et al.*, 2012) with and without *GBA* mutations. Current evidence thus supports a central role for neuronal loss of glucosylceramidase activity in the pathogenesis of neurodegeneration associated with *GBA* mutations. The majority of *GBA* mutations do not affect the catalytic site of glucosylceramidase, and so other mechanisms must account for loss of enzyme activity in Parkinson’s disease and Gaucher disease (Hruska *et al.*, 2008). There is evidence that *GBA* missense mutations cause endoplasmic reticulum retention and proteasomal degradation of the mutant protein (Ron *et al.*, 2005; Sawkar *et al.*, 2006). Given that there is reduction of glucosylceramidase activity in brain tissue from patients with Parkinson’s disease and those with Lewy body dementia, with and without *GBA* mutations, there is clearly an urgent need to understand how reduced glucosylceramidase activity causes neurodegeneration to facilitate development of new treatments.

The *GBA* gene is part of the CLEAR network (coordinated lysosomal expression and regulation), which consists of >400 genes (Palmieri *et al.*, 2011). The CLEAR network is regulated by transcription factor EB (TFEB), which acts to induce transcriptional upregulation of the CLEAR network in a synchronized fashion (Song *et al.*, 2013). The genes of the CLEAR network encode all the proteins required for lysosomal biogenesis and function; including the lysosomal enzymes, lysosomal membrane proteins, autophagy molecules and proteins required for trafficking. The CLEAR network thus represents an attractive therapeutic target for improving function of the autophagy–lysosome system in Parkinson’s disease. Demonstrating activation of the CLEAR network can be performed by showing synchronous upregulation of CLEAR network genes by using microarray gene expression profiling (Palmieri *et al.*, 2011).

Here we focused on identifying cellular biochemical changes associated with *GBA* mutations that might predispose to neurodegeneration using fibroblasts generated from a series of patients with Gaucher disease and heterozygous *GBA* mutation carriers with and without Parkinson’s disease. We then reasoned that ambroxol hydrochloride, a small molecule previously shown to improve glucosylceramidase function in Gaucher disease (Maegawa *et al.*, 2009), might ameliorate the cellular phenotypes in Gaucher disease and *GBA* mutation heterozygotes. Our findings indicate that *GBA* mutations are associated with oxidative and endoplasmic reticulum stress and that ambroxol can improve lysosomal biochemistry in these cells by activating the CLEAR network. We propose that these results are of relevance to the targeting of potential novel interventions to slow the progression of Parkinson’s disease and other *GBA*-related disorders.

## Materials and methods

### Patients and fibroblast culture

Fibroblasts were generated from skin biopsies of five patients with type I Gaucher disease, four heterozygous carriers with Parkinson’s disease, one patient with Parkinson’s disease with homozygous E326K *GBA* mutation, two carriers without Parkinson’s disease (non-manifesting carriers) and three patients with Parkinson’s disease without *GBA* mutations. For comparison, fibroblasts were generated from three control subjects without neurological disease or *GBA* mutations. All groups were matched for age and sex. The clinical characteristics and mutation spectrum of the patient cohort are given in Table 1. Fibroblasts were grown in Dulbecco’s modified Eagle medium with 4.5 g/l glucose (Invitrogen), 5% serum, 5% pyruvate, fungizone and penicillin–streptomycin. Fibroblasts were studied at low passage, and disease and control were matched for passage number.

**Table 1:** Characteristics of patient-derived fibroblasts

Patient (ID, gender/age)	Genotype	Clinical features
**Gaucher disease**		
GD01, M/50	N370S/c1263del55	Type I Gaucher disease
GD02, M/70	N370S/N370S	Type I Gaucher disease
GD03, M/55	N370S/L444P	Type I Gaucher disease
GD04, M/61	N370S/203insC	Type I Gaucher disease
GD05, M/40	N370S/L444P	Type I Gaucher disease
**Non-manifesting carrier**		
C01, F/80	N370S/WT	No neurological disease
C02, M/62	L444P/WT	No neurological disease
**Parkinson’s disease with *GBA* mutation**		
PD01, F/53	N370S/WT	Parkinson’s disease
PD02, M/45	N370S/WT	Parkinson’s disease
PD03, M/72	L444P/WT	Parkinson’s disease
PD04, F/61	RecNcil/WT	Parkinson’s disease
PD05, M/50	E326K/E326K	Parkinson’s disease
**Control**		
CN01, M/78	WT/WT	No neurological disease
CN02, M/81	WT/WT	No neurological disease
CN03, F/50	WT/WT	No neurological disease

WT = wild-type.

### Alpha-synuclein over expressing cell lines

To investigate factors modulating cellular alpha-synuclein levels, an SH-SY5Y stable cell line expressing high levels of exogenous wild-type alpha-synuclein were used. Exogenously expressed alpha-synuclein contains a haemagglutinin tag (HA) at the C-terminal. All SH-SY5Y cell lines were cultured in 1:1 Dulbecco’s modified Eagle medium/F12 (Invitrogen) supplemented with 10% foetal calf serum, non-essential amino acids, 1 mM sodium pyruvate and penicillin–streptomycin.

### Indirect immunofluorescence

Cells were grown on coverslips, fixed in 4% paraformaldehyde for 15 min and permeabilized in ice cold methanol. Blocking was performed in 2% goat serum before incubation with primary and secondary antibodies. Anti-glucocerebrosidase (Abcam) was used at a concentration of 1:50 and anti-calnexin (Abcam) at 1:500.

### Lysosomal enzyme assays

The glucosylceramidase enzymatic assay was performed using a fluorometric assay with 10 mM 4-methylumbelliferyl-β-d-glucopyranoside (Sigma) as the substrate. Glucosylceramidase activity is reported in the presence of the activator sodium taurocholate (Sigma). Total beta-hexosaminidase and beta-galactosidase were assayed with 4-methylumbelliferyl-2-acetoamido-2-deoxy-6-sulpho-b-d-glucopyransoside and 4-methylumbelliferyl-b-d-galactopyranoside as substrates, respectively.

### Lyso-ID® protocol

Cells were grown overnight in 96-well plates, three wells per cell line. Cells were incubated for 30 min with Lyso-ID green dye (Enzo life sciences), a cell permeable fluorescent probe which localizes to acidic vesicles (endosomes, lysosomes, late autophagosomes). Dye was washed off and fluorescence read on a plate reader, with results normalized to protein content.

### Western blotting

Fibroblasts were trypsinized and resuspended in 1 ml PBS. Each sample was then treated with 100 µl lysis buffer with protease inhibitors (1% Triton™ X-100 with 1 µg/ml leupeptin, 1 µM PMSF, 1 µg/ml pepstatin, 1 μM NaV). Cell lysates were then centrifuged at full speed for 10 min and the supernatant transferred to fresh tubes. Lysates containing 30 µg of protein were then electrophoresed on a 4–12% NuPage gel (Invitrogen). Proteins were transferred to a PVDF membrane (Amersham), blocked in non-fat milk, treated with primary antibody and secondary antibodies. Antibody binding was then detected using an ECL chemiluminescence kit. The following antibodies were used: glucocerebrosidase (ab55080 Abcam), actin (AC15 Abcam), LIMP2 (ab106519 Abcam), LC-3 (Cell signalling), cathepsin D (ab40697 Abcam), anti-haemagglutin (covance HA.11) and p62 (ab56416 Abcam). For each antibody, three controls were run alongside the patient samples on each gel. The band intensity for the three controls was averaged and the patient results were expressed as a percentage of the mean control value.

### TFEB enzyme-linked immunosorbent assay

The TFEB ELISA was developed and performed in-house at Signosis Inc. Nuclear and cytosolic fractionation of fibroblasts was performed in-house at Signosis Inc. using the SK-0001 nuclear extraction kit. The TFEB ELISA was performed on three separate cultures from control line 3 treated with ambroxol and three separate untreated cultures from control line 3.

### Endoglycosidase-H treatment

Twenty micrograms of protein was denatured with glycoprotein denaturing buffer (New England Biolabs 5% SDS, 0.4 M dithiothreitol) at 100°C. The lysate was then incubated for 12 h with 1 000 units of endoglycosidase-H and reaction buffer (New England Biolabs 0.5 M sodium citrate, pH 5.5). The lysate was then used in the western blot protocol outlined above.

### Proteasome inhibition

Sub-confluent 10-cm plates of fibroblasts were treated with proteasome inhibitor (MG132 15 μM and ALLN 25 μM, sigma) for 18 h. This cocktail significantly inhibits PGP-like (MG132 100% and ALLN 92% inhibition, *P < *0.05), chymotrypsin (53% and 57% inhibition, *P < *0.05) and trypsin-like (54% and 65% inhibition, *P < *0.05) activity in neuroblastoma cells.

### Ambroxol hydrochloride treatment

An initial titration experiment was performed to select the dose of ambroxol hydrochloride to use for rescue experiments. Ambroxol hydrochloride (Sigma) was dissolved in dimethylsulphoxide (DMSO) and diluted in cell culture media to give a final concentration of 10 µM, 30 µM and 60 µM. This dose range was selected based upon dosages used in previous *in vitro* studies (Maegawa *et al.*, 2009; Luan *et al.*, 2013). In the initial titration experiment a control line and a Gaucher disease line were treated with 10 µM, 30 µM and 60 µM for 5 days. Media were changed daily for ambroxol treated and control lines. Controls were treated with DMSO only. The greatest increase in glucosylceramidase protein levels were observed with 60 µM (Supplementary Fig. 1), and this dose was therefore selected for the rescue experiments.

### Gene expression profiling

RNA was extracted from cultured fibroblasts (<10^6^ cells) using the RNeasy® kit (Qiagen) and converted to complementary DNA with QuantiTect reverse transcription kit (Qiagen). Gene expression profiling was performed on complementary DNA from a single control fibroblast line with (*n = *3) and without (*n = *3) ambroxol treatment using a GeneChip Human Genome U133 Plus 2.0 Array from Affymetrix. Control fibroblasts were used to avoid potential confounding as a result of changes in gene expression potentially associated with *GBA* mutations. Hybridizations and data analyses were performed at Source Bioscience. Gene set enrichment analysis was performed as described previously for lysosome and autophagy gene sets (Subramanian *et al.*, 2005). A nominal *P*-value <0.05 and a false discovery rate of <2% were used to assess the statistical significance of the enrichment score. Gene set ANOVA was performed on KEGG pathways (lysosome, autophagy) using Partek Genomics Suite (6.5). Relative expression of *GBA*, *NQO1* and β-actin messenger RNA in untreated cells was measured with Power SYBR® Green kit (Applied Biosystems) using a StepOne™ PCR machine (Applied Biosystems). To analyse the effect of ambroxol treatment both treated and untreated cells had TFEB, aspartyl glucosaminidase, cathepsin K, SCARB2/LIMP2, hexosaminidase A and iduronidase alpha measured using the TaqMan® PCR system. β-actin messenger RNA levels were used to normalise data. Relative expression was calculated using the ΔC_T_ method. Details of primers are given in Supplementary Table 1.

### Single cell imaging of dihydroethidium and monochlorobimane

Cytosolic reactive oxygen species production was monitored by single cell analysis of dihydroethidium oxidation rate. For each cell line, fibroblasts were imaged with dihydroethidium dissolved in HEPES buffered salt solution (156 mM NaCl, 3 mM KCl, 2 mM MgSO_4_, 1.25 mM KH_2_PO_4_, 2 mM CaCl_2_, 10 mM glucose, and 10 mM HEPES, pH adjusted to 7.35 with NaOH). No preincubation (‘loading’) was performed to minimize intracellular accumulation of the oxidized product. The amount of dihydroethidium used in each run was determined by adjusting the concentration of dihydroethidium until it was sufficient to ensure that cellular non-oxidized (i.e. cytoplasmic) fluorescence reached a peak rapidly. Fluorescence images were obtained on an epifluorescence inverted microscope equipped with a ×20 fluorite objective (Cairn Research). For hydroethidium measurements, a ratio of the oxidized to reduced form was measured: excitation at 530 nm and emission recorded >560 nm were used to quantify the oxidized form (ethidium), whereas excitation at 360 nm and emission collected from 405 to 470 nm was used for the reduced form (dihydroethidium). All data reported in this study were obtained from at least five coverslips and two to three different cell and sample preparations.

### Glutathione measurements

To measure glutathione (GSH), cells were incubated with 50 µM monochlorobimane (Molecular Probes). As monochlorobimane reacts with GSH in a reaction catalysed by gluthatione S-transferase, generating a fluorescent adduct, cells were incubated with the dye in HEPES balanced salt solution at room temperature for 40 min, or until a steady state had been reached before images were acquired for quantitation. The cells were then washed with HEPES balanced salt solution, and images of the fluorescence of the monochlorobimane-GSH adduct were acquired using the LSM510 system using excitation at 380 nm and emission at >400 nm.

### Statistical and image analysis

Densitometric analysis of western blots was performed using ImageJ (NIH, http://rsb.info.nih.gov/ij/). Statistical analysis was performed using PASW (IBM version 20.1). Means were compared with Student’s *t*-test and medians with a Mann-Whitney U-test. Medians were used to compare non-parametric data (percentages of protein expression on western blot and fold change of protein and transcript levels), means were used to compare parametric data (rates of enzyme activity). For between-group comparisons (Gaucher disease, E326k/E326K, Parkinson’s disease or non-manifesting carrier compared with controls) Bonferroni correction was applied. As four comparisons were being made for each marker significance at the 5% level was set at *P* = 0.0125 (0.05/4). Bonferroni correction was not applied to comparisons made between cells treated with ambroxol and their untreated control, as this was a single statistical test and multiple comparisons were not made. The statistical methodology for the gene expression profiling experiments is detailed above. All experiments were performed in biological triplicate.

## Results

Glucosylceramidase activity is decreased in fibroblasts from patients with Gaucher disease and heterozygous *GBA* mutation carriers with and without Parkinson’s disease

**Figure 1:**
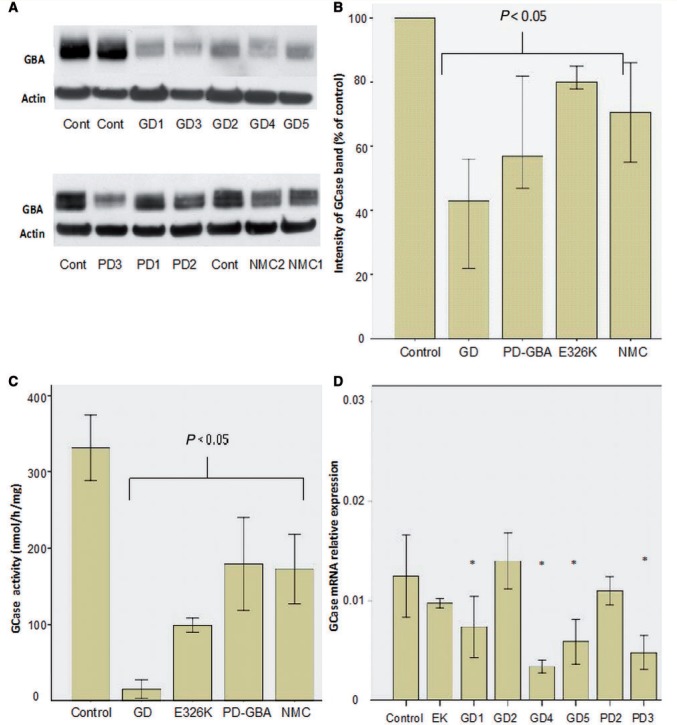
Glucosylceramidase protein, activity and transcript levels. (**A**) Representative western blots of glucosylceramidase protein. Note reduction in glucosylceramidase protein levels in Gaucher disease (GD) fibroblasts with a lesser degree of reduction in Parkinson’s disease with glucocerebrosidase mutations (PD-GBA), non-manifesting carrier (NMC) and the E326K/E326K fibroblasts. (**B**) Bar chart summarizing glucosylceramidase protein levels as assessed by western blot normalized to control. Bars represent median value and 95% confidence interval for all cell lines in each group; each line was measured on three separate blots. There are significant reductions in Gaucher disease (Mann-Whitney U-test *P < *0.001), Parkinson’s disease with glucocerebrosidase mutations (*P < *0.001), non-manifesting carrier (*P = *0.004) and E326K/E326K lines (*P = *0.003). (**C**) Bar chart summarizing glucosylceramidase activity levels (nmol/h/mg). Enzyme activity was significantly reduced in fibroblasts from patients with Gaucher disease (*t*-test *P = *0.001), patients with Parkinson’s disease with glucocerebrosidase mutations (*P = *0.004), non-manifesting carriers (*P = *0.009) and E326k/E326K lines (*P = *0.001) compared with controls. Each bar represents all cell lines in each group. Results are mean ± 1 SD of three separate experiments done for each line. (**D**) GBA transcript levels in disease lines compared to controls (*n = *3), there were reduced transcript levels in GD01, GD04, GD05, and PD03. *P < 0.05.

Western blotting revealed a significant reduction in glucosylceramidase protein levels in all Gaucher disease, Parkinson’s disease with *GBA* mutation, E326K/E326K and non-manifesting carrier cell lines (Fig. 1A and B). Glucosylceramidase activity (measured in the presence of sodium taurocholate) in Gaucher disease, Parkinson’s disease with *GBA* mutation and non-manifesting carrier lines was significantly lower than controls (Fig. 1C). Glucosylceramidase activity in fibroblasts from the E326K homozygous Parkinson’s disease case was also significantly reduced compared with controls, at a level intermediate between Gaucher disease, Parkinson’s disease with *GBA* mutation and non-manifesting carrier. Glucosylceramidase activity in fibroblasts from patients with sporadic Parkinson’s disease did not differ from controls (365 ± 30 versus 325 ± 30 nmol/mg/h, *t*-test *P = *0.17).

*GBA* transcript levels were reduced in Gaucher disease lines with N370S/203insC (Patient GD4, median 30% of control, Mann-Whitney U test *P = *0.001), N370S/c1263del55 (Patient GD1, median 66% of control, *P = *0.007) or N370S/L444P (Patient GD5 median 56% of control, *P = *0.001) mutations and in Parkinson’s disease with L444P (Patient PD3 median 42% of control, *P = *0.001) or RecNcil (Patient PD4, 55% of control, *P = *0.007) mutations (Fig. 1). Transcript levels were not reduced in N370S/N3270S Gaucher disease (Patient GD2), E326K homozygous cells or Parkinson’s disease fibroblasts with N370S alleles (Patients PD1 and PD2) (Fig. 1D).

LIMP2 (*SCARB2*) acts as a chaperone helping to traffic glucosylceramidase from the endoplasmic reticulum to the lysosome. To ensure that glucosylceramidase depletion was not secondary to loss of LIMP2 expression western blot measurement of LIMP2 levels was performed. LIMP2 levels did not differ from controls in either Gaucher disease {median 93% of control [interquartile range (IQR) 90–105%], Mann-Whitney U-test *P = *0.33}, Parkinson’s disease with *GBA* mutations [median 93% of control (IQR 90–100%), *P = *0.12] or non-manifesting carrier [median 95% (IQR 90–105%) *P = *0.1] fibroblasts (data not shown).

**Figure 2:**
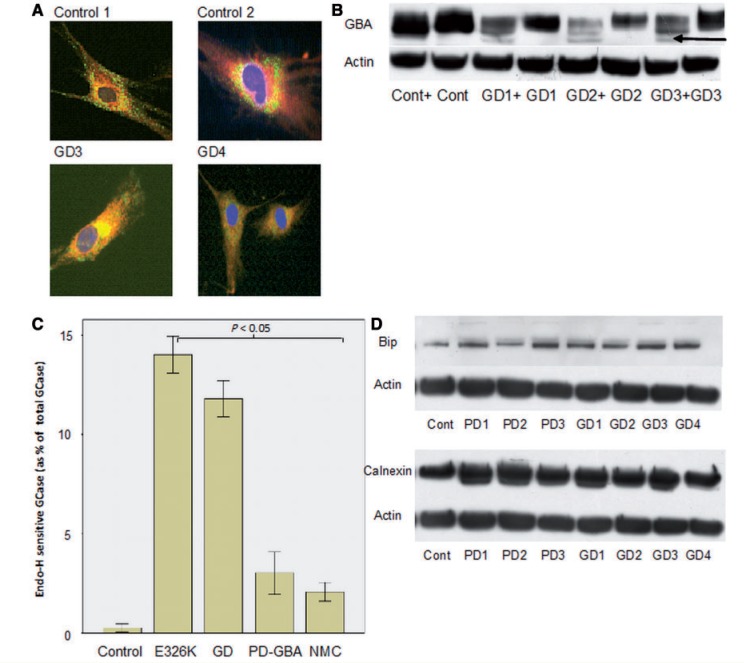
Evidence of endoplasmic reticulum retention of glucosylceramidase in Gaucher disease. (**A**) *Top*: immunofluorescence of control cells showing vesicular staining of glucosylceramidase (green) at the periphery of the cell and perinuclear, reticular staining of calnexin (red, endoplasmic reticulum marker). *Bottom*: co-localization of glucosylceramidase and calnexin represented by yellow perinuclear staining with minimal green vesicular staining pattern in GD02 and GD03 cell lines. (**B**) Representative western blots of cell lysate treated with endoglycosidase-H for 12 h. Note appearance of low molecular weight band in Gaucher disease samples (arrow), which represents endoplasmic reticulum retained glucosylceramidase protein. ‘+’ lanes were treated with endoglycosidase-H. (**C**) Bar chart summarizing percentage of endoglycosidase-H sensitive glucosylceramidase in Gaucher disease (GD) (*t*-test *P = *0.0001), Parkinson’s disease with glucocerebrosidase mutations (PD-GBA) (*P = *0.01), non-manifesting carriers (NMC) and E326K/E326K (*P = *0.001) compared with controls. Results are mean of three experiments. (**D**) *Top*: representative western blot of BiP expression. *Bottom*: representative western blot of calnexin expression. There was significant elevation of endoplasmic reticulum stress markers BiP and calnexin expression in all Gaucher disease, Parkinson’s disease with *GBA* mutation and non-manifesting carrier lines.

For each cell line indirect immunofluorescence using antibodies to glucosylceramidase and calnexin (an endoplasmic reticulum marker) was performed. In control cells glucosylceramidase was clearly seen to accumulate in punctate structures at the cell periphery which morphologically resembled lysosomes and did not co-localize with calnexin. The distribution of glucosylceramidase in Parkinson’s disease with *GBA* mutation and non-manifesting carrier cells was not readily distinguishable from control fibroblast lines. However, in Gaucher disease fibroblasts there was a marked reduction in the staining of the punctate lysosomal structures, most of the glucosylceramidase was observed to accumulate in a peri-nuclear pattern and co-localize with calnexin (yellow perinuclear staining in cells in Fig. 2A). Endoplasmic reticulum retained forms of glucosylceramidase carry *N*-linked glycans which are sensitive to cleavage by endoglycosidase-H, producing a low molecular weight band of glycosylceramidase on western blotting. The endoglycosidase-H sensitive fraction of glucosylceramidase was significantly higher in Gaucher disease, E326K/E326K, Parkinson’s disease with *GBA* mutation and non-manifesting carrier fibroblasts; indicating endoplasmic reticulum retention in all these lines (Fig. 2B and C).

Given the reduced glucosylceramidase protein levels and evidence of endoplasmic reticulum retention of glucosylceramidase in the disease fibroblasts, we hypothesized that reduced levels may result from proteasomal degradation. Disease and control fibroblasts were treated with the proteasomal inhibitors MG132 and ALLN for 18 h and then harvested. Proteasome inhibition resulted in elevated glucosylceramidase protein levels, as assessed by western blotting, within Gaucher disease fibroblasts [median 49% increase (IQR 30–62%), Mann-Whitney U-test *P = *0.029] and Parkinson’s disease with *GBA* mutation fibroblasts [median increase 25% (IQR 15–35%), *P = *0.043] to a greater degree than in controls (median 2% increase).

### *GBA* mutations are associated with markers of endoplasmic reticulum and oxidative stress in fibroblasts

Given the evidence of endoplasmic reticulum retention of glucosylceramidase we assessed the cell lines for evidence of endoplasmic reticulum stress by western blotting (Fig. 2D). There was BiP/Grp78 elevation on western blotting of Gaucher disease [median 60% increase compared with controls (IQR 20–100% increase), Mann-Whitney U-test *P = *0.0016], Parkinson’s disease with *GBA* mutation [median 15% increase (IQR 0–50%), *P = *0.0023] and non-manifesting carrier cells [median 15% increase (IQR 10–20%), *P = *0.009]. Calnexin levels were significantly elevated in Gaucher disease fibroblasts [median 50% increase (IQR 50–100%), Mann-Whitney U-test *P = *0.003] and Parkinson’s disease with *GBA* mutation fibroblasts [median 32% increase (IQR 12–50%), *P = *0.019].

**Figure 3:**
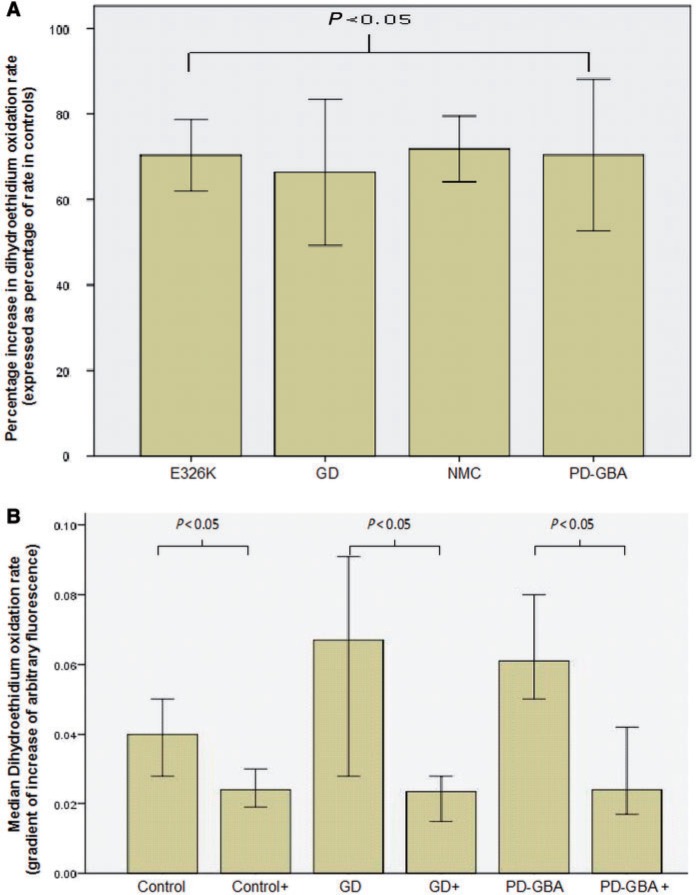
Oxidative stress assays in Gaucher and Parkinson’s disease fibroblasts. (**A**) Graph summarizing increases in rates of dihydroethidium oxidation rates for Gaucher disease (GD) (Mann-Whitney U-test *P < *0.001), Parkinson’s disease with glucocerebrosidase mutations (PD-GBA) (*P < *0.001), non-manifesting carriers (NMC) (*P < *0.001) and E326K/E326K (*P < *0.001) compared with control cells. (**B**) Graph demonstrating significant reduction in dihydroethidium oxidation rates for controls (*n = *2), Gaucher disease (*n = *3) and Parkinson’s disease with glucocerebrosidase mutations fibroblasts (*n = *2) treated with ambroxol compared to untreated cells. Results expressed as median rate of dihydroethidium oxidation ± 95% confidence interval.

We next measured basal oxidative stress. The rate of dihydroethidium oxidation was significantly elevated in Gaucher disease, Parkinson’s disease with *GBA* mutation, non-manifesting carrier and the E326K/E326K fibroblast lines (Fig. 3A). The intensity of monochlorobimane staining of Gaucher disease [median 77% of control values (IQR 50–90%), Mann-Whitney U-test *P = *0.001] and Parkinson’s disease with *GBA* mutation fibroblasts [median 72% (IQR 50–92%) of control, *P = *0.001] was significantly less than that of control fibroblasts. This suggests a reduction in cellular glutathione levels. Transcript levels of the anti-oxidant NQO1 were significantly elevated in Gaucher disease [0.017 (IQR 0.014–0.019), Mann-Whitney U-test *P = *0.012] and Parkinson’s disease with *GBA* mutation fibroblasts [0.02 (0.016–0.023), *P = *0.024] compared with controls [0.01 (IQR 0.007–0.014)].

### *GBA* mutations cause alterations of lysosomal biochemistry in fibroblasts

**Figure 4:**
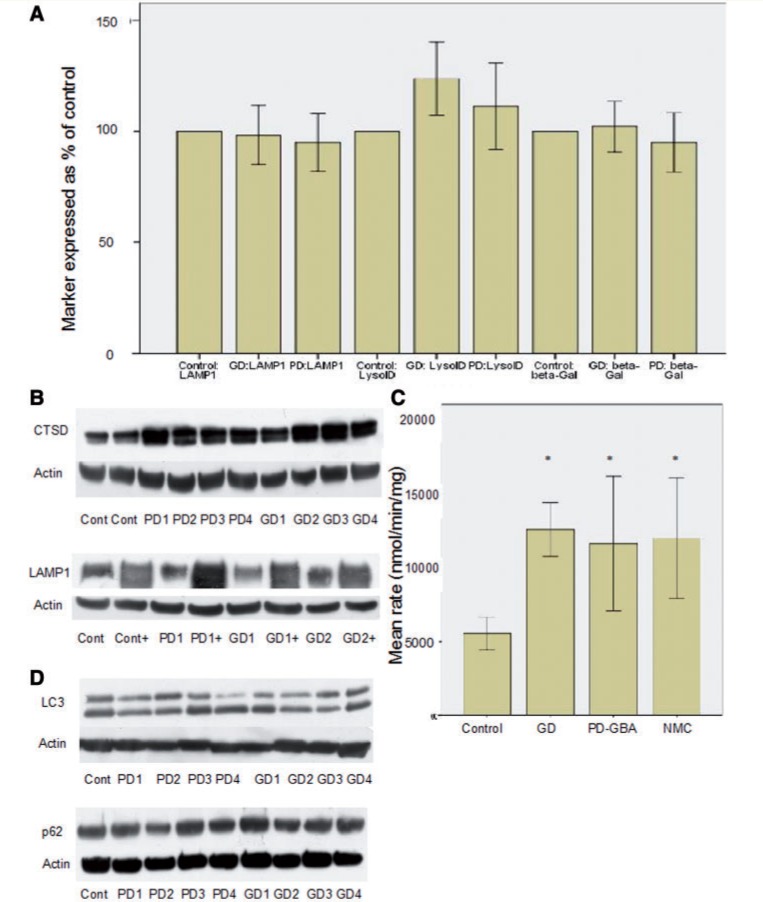
Analysis of lysosomal markers and autophagy. (**A**) Bar chart summarizing results for LAMP1 western blot, LysoID assay and beta-galactosidase activity. There is no significant difference in any of these variables, suggesting lysosomal mass is not altered. For each variable results are expressed as a percentage of results from control cells (*n = *3). (**B**) Representative western blots demonstrating increased cathepsin D in Gaucher disease and Parkinson’s disease with *GBA* mutations compared to controls (*top* panel) and increased LAMP1 expression after ambroxol treatment (lanes marked ‘+’, *bottom* panel). (**C**) Bar chart summarizing increase in total beta-hexosaminidase activity in Gaucher disease (GD), Parkinson’s disease with *GBA* mutations (PD-GBA) and non-manifesting carriers (NMC) compared with controls. *P < 0.05. (**D**) Represenative western blots demonstrating LC3-II levels (*top*) and p62 (*bottom*). There was no significant difference in either marker in Gaucher disease or Parkinson’s disease with *GBA* mutations compared to control.

There is no alteration in lysosomal mass in Gaucher disease or Parkinson’s disease with *GBA* mutation fibroblasts; the number of acidic vesicles (Lyso-ID), LAMP1 protein levels and beta-galactosidase activity does not differ between Gaucher disease, Parkinson’s disease with *GBA* mutation, non-manifesting carrier and control (Fig. 4A). On western blotting, cathepsin D was significantly increased in Gaucher disease [median 50% increase (IQR 32–100%), Mann-Whitney U-test *P < *0.0001] and Parkinson’s disease with *GBA* mutation [median 30% increase (IQR 20–40%), *P = *0.002] fibroblasts (Fig. 4D). Total beta-hexosaminidase activity was elevated in Gaucher disease, Parkinson’s disease with *GBA* mutation and non-manifesting carriers compared with controls (Fig. 4B). Together, these data suggest secondary changes in cathepsin D and total beta-hexosaminidase metabolism that are not because of an overall expansion of the lysosomal compartment.

Given the evidence of potential lysosomal dysfunction we investigated macroautophagy (Fig. 4D). Under basal conditions there was no elevation of p62 in Gaucher disease [median 101% (IQR 90–110%) of control, Mann-Whitney U-test *P = *0.9], Parkinson’s disease with *GBA* mutation fibroblasts [median 100% (IQR 95–105%) of control, *P = *0.8] or non-manifesting carriers (median 99% of control, *P = *0.8). Without starvation there was no alteration of LC3-II in Gaucher disease (mean 82 ± 20% of control, *P = *0.062), Parkinson’s disease with *GBA* mutation (mean 88 ± 15% of control, *P = *0.13) or non-manifesting carriers (99% of control, *P = *0.8). This suggests that macroautophagy is not abnormal.

### Ambroxol hydrochloride improves glucosylceramidase activity in fibroblasts with *GBA* mutations by activating the CLEAR network

**Figure 5:**
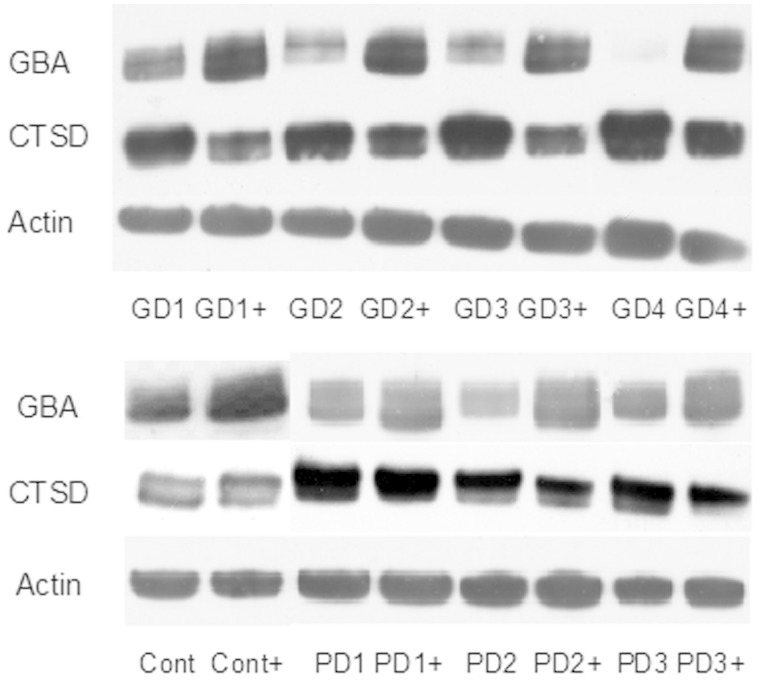
Ambroxol treatment significantly increases glucosylceramidase protein levels. Representative western blots showing increased glucosylceramidase protein levels and decreased cathepsin D levels with ambroxol treatment. ‘+’ represents lanes treated with ambroxol. Ambroxol treatment resulted in a significant elevation of glucosylceramidase protein levels in control [median increase 30% of untreated cells (IQR 15–40%), Mann-Whitney U-test *P = *0.0085], Gaucher disease (GD) [median increase 100% of untreated (IQR 87–200% increase), *P = *0.004], Parkinson’s disease with *GBA* mutations (PD-GBA) [median increase 50% of untreated (IQR 40–130%), *P = *0.04] and non-manifesting carrier (NMC) fibroblasts [median increase 35% of untreated cells (IQR 25–40% increase), *P = *0.008]. Results are derived from all cell lines in each group and represent median value and IQR.

**Figure 6:**
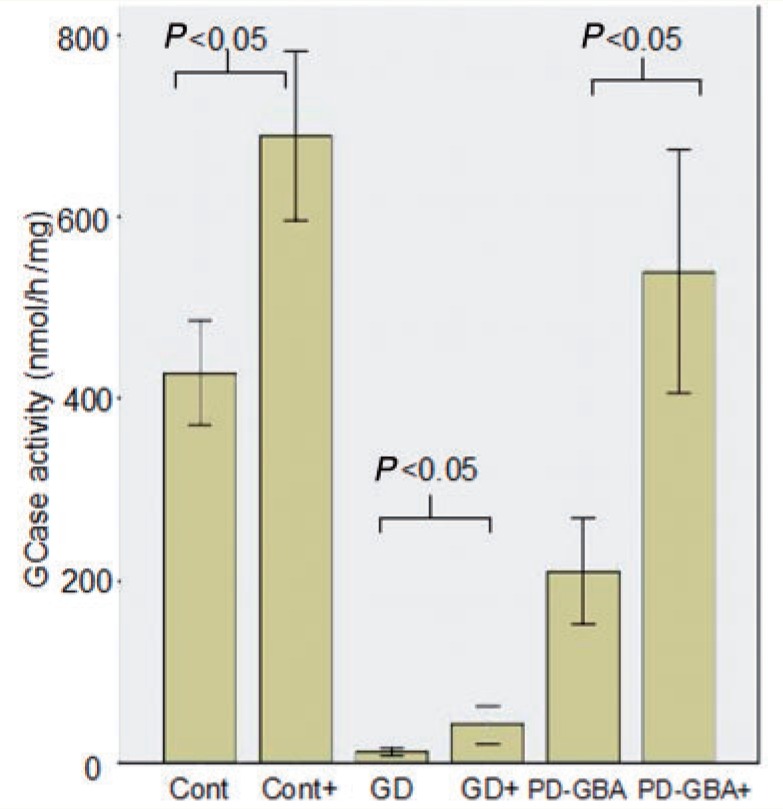
Ambroxol treatment significantly increases glucosylceramidase enzyme activity. Bar chart demonstrating significant increase in glucosylceramidase activity (nmol/mg/h) with ambroxol treatment in controls (*t*-test *P = *0.001), Gaucher disease (GD) (*P = *0.0001) and Parkinson’s disease with glucocerebrosidase mutations (PD-GBA) (*P = *0.0001). ‘+’ = with ambroxol treatment. Results are derived from all cell lines in each group and represent the mean of three experiments ± 1 standard deviation.

**Figure 7:**
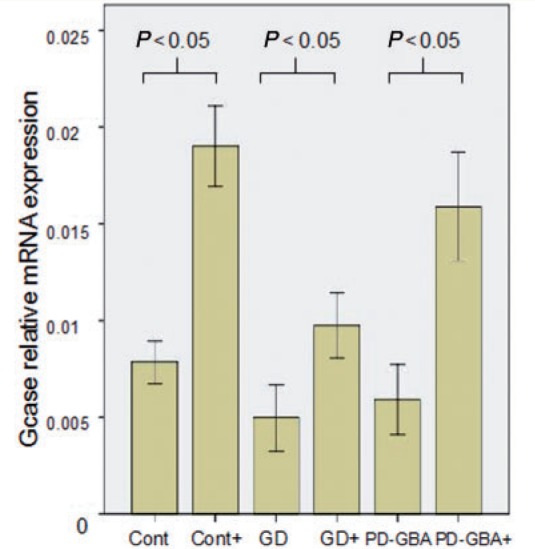
Ambroxol treatment significantly increases glucosylceramidase transcript levels. Bar chart demonstrating significant increase in glucosylceramidase (GCase) messenger RNA levels after ambroxol treatment in controls (Mann-Whitney U-test, *P = *0.001), Gaucher disease (GD) (*P = *0.0015) and Parkinson’s disease with glucocerebrosidase mutations (PD-GBA) (*P = *0.001). Results are median of three experiments ± 95% confidence interval.

Ambroxol treatment resulted in a significant elevation of glucosylceramidase protein levels in control [median increase 30% of untreated cells (IQR 15–40%), Mann-Whitney U-test *P = *0.0085], Gaucher disease [median increase 100% of untreated (IQR 87–200% increase), *P = *0.004], Parkinson’s disease with *GBA* mutations [median increase 50% of untreated (IQR 40–130%), *P = *0.04] and non-manifesting carrier fibroblasts [median increase 35% of untreated cells (IQR 25–40% increase), *P = *0.008] on western blotting (Fig. 5). Ambroxol treatment also resulted in a significant elevation of glucosylceramidase activity levels (Fig. 6) and messenger RNA (Fig. 7) in Gaucher disease, Parkinson’s disease with *GBA* mutations and non-manifesting carriers. This was accompanied by an almost complete abolition of the endoglycosidase-H sensitive glucosylceramidase fraction (Fig. 9A) in Gaucher disease fibroblasts. There was no evidence of cell death after ambroxol treatment of control or Gaucher disease fibroblasts [median decrease of 15% fluorescent units (FU)/5 000 cells treated versus median decrease of 11% of FU/5 000 cells treated, Mann-Whitney U-test *P = *0.96].

To determine whether ambroxol treatment for 5 days increased glucosylceramidase expression by activating the CLEAR network, we performed gene expression microarray analysis of treated and untreated control fibroblasts with a GeneChip Human Genome U133 Plus 2.0 Array. We used control fibroblasts to avoid any potential confounding caused by effects of *GBA* mutations on lysosomal gene expression. Gene set enrichment analysis examines how a subset of genes of interest is distributed through a set of differentially expressed genes, by ranking genes from most upregulated to most downregulated (Subramanian *et al.*, 2005). An enrichment score is then calculated to summarize the tendency of the genes of interest to be upregulated (enrichment score from 0 to +1) or downregulated enrichment score from (0 to −1). Gene set enrichment analysis demonstrated that the majority of genes involved in lysosomal metabolism (enrichment score +0.54, *P = *0.04, false discovery rate 0.02) or autophagy (enrichment score +0.66, *P = *0.04, false discovery rate 0.022) were upregulated. This result was supported by a gene set ANOVA based on KEGG pathways demonstrated that the lysosomal pathway was upregulated 1.21-fold in treated cells (*P = *4.85 × 10^−5^) compared with untreated cells. Supplementary Table 2 details the genes involved in lysosomal function and autophagy upregulated after ambroxol treatment.

To confirm these microarray results we examined the induction of a subset of CLEAR genes after ambroxol treatment using quantitative PCR, western blotting and enzymatic assays. Transcripts for the lysosomal proteins hexosaminidase subunit A, aspartyl glucosaminidase, cathepsin K, SCARB2/LIMP2 and iduronidase alpha all rose significantly in control, Gaucher disease and Parkinson’s disease with *GBA* mutation cells after ambroxol treatment (Supplementary Table 3). On western blotting LAMP1 levels rose significantly in controls, Gaucher disease and Parkinson’s disease with *GBA* mutation after ambroxol treatment (median increase 100% in each group, *P < *0.05, Fig. 4C). Enzymatic activity of beta-galactosidase rose significantly after ambroxol treatment in control [median 60% increase (IQR 50–60%), Mann-Whitney U-test *P = *0.035], Gaucher disease [median 50% increase (IQR 45–100%), *P = *0.04] and Parkinson’s disease with *GBA* mutation [median 70% increase (IQR 36–100%), *P = *0.03].

**Figure 8:**
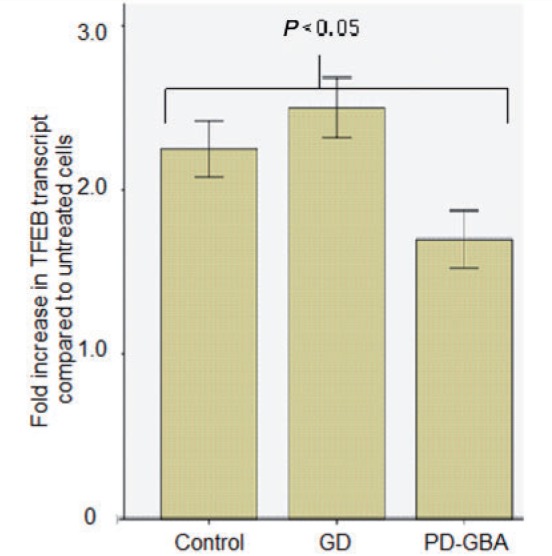
Ambroxol significantly increases TFEB transcript levels. Bar chart summarizing increase (fold increase) in TFEB transcript levels in cells treated with ambroxol, data are from two cell lines in each group. There was a significant increase in TFEB transcript levels for control (Mann-Whitney U-test *P = *0.02), Gaucher disease (GD) (*P = *0.01) and Parkinson’s disease with glucocerebrosidase mutations (PD-GBA) (*P = *0.03) after treatment with ambroxol.

The transcription factor TFEB activates the CLEAR network to control lysosomal biogenesis. TFEB transcript levels rose significantly after ambroxol treatment in control (2.25-fold increase, Mann-Whitney U-test *P = *0.02), Gaucher disease (2.5-fold increase, *P = *0.01) and Parkinson’s disease with *GBA* mutation fibroblasts (1.75-fold increase, *P = *0.03) (Fig. 8). TFEB strongly induces its own expression; therefore upregulation of TFEB along with evidence of activation of the CLEAR network strongly suggests activation of TFEB transcriptional activity. To confirm this we quantified nuclear TFEB expression using an ELISA in control fibroblasts treated with ambroxol. Nuclear localization of TFEB increased in control fibroblasts treated with ambroxol compared to untreated cells [63% (IQR 63–65%) versus 51% (IQR 50–52%) in untreated, Mann-Whitney U-test *P = *0.046].

### Ambroxol treatment improves lysosomal biochemistry and oxidative stress in fibroblasts carrying *GBA* mutations

In control cells cathepsin D levels did not change and beta-hexosaminidase activity was elevated [median 15% (IQR 5–15%), Mann-Whitney U-test *P = *0.04] after ambroxol treatment. After ambroxol treatment cathepsin D levels fell in Gaucher disease [median fall 30% (IQR 25–50%), *P = *0.03] and Parkinson’s disease with *GBA* mutation cells [median fall 20% (IQR 15–40%), *P = *0.04] (Fig. 5), as did total beta-hexosaminidase activity [Gaucher disease median fall 36.5% (IQR 26.2–50%), *P = *0.012 and Parkinson’s disease with *GBA* mutation median fall 30% (IQR 20–50%), *P = *0.019]. This suggests that ambroxol is inducing a general increase in lysosomal mass, with improvement of glucosylceramidase activity in Gaucher disease, Parkinson’s disease with *GBA* mutations and non-manifesting carriers resulting in a fall in cathepsin D and beta-hexosaminidase activity.

Ambroxol treatment significantly reduced dihydroethidium oxidation rate in Gaucher disease [median dihydroethidium oxidation rate ΔRFU 0.05 (0.028–0.11) versus 0.027 (0.014–0.037) with ambroxol, Mann-Whitney U-test *P = *0.004], Parkinson’s disease with *GBA* mutation [ΔRFU 0.06 (0.04–0.8) versus 0.03 (0.017–0.044), *P = *0.001] and control [ΔRFU 0.04 (0.024–0.06) versus 0.026 (0.02–0.03), *P* = 0.028] lines (Fig. 3B).

### Ambroxol treatment reduces alpha-synuclein levels in an overexpressing neuroblastoma line

**Figure 9:**
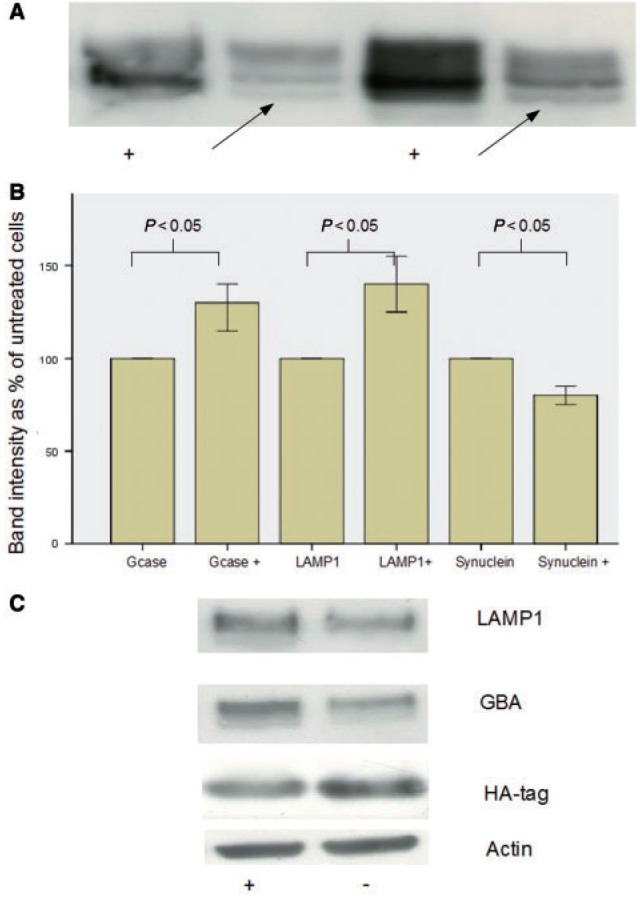
Effect of ambroxol on alpha-synuclein overexpressing neuroblastoma cell lines. (**A**) Representative western blots demonstrating disappearance of endoglycosidase-H sensitive band (arrow) in ambroxol treated Gaucher disease cell lines. ‘+’ = treated with ambroxol and the arrow indicates the endoglycosidase-H sensitive fraction. (**B**) Bar chart summarizing significant increase in glucosylceramidase and LAMP1 and significant decrease in alpha-synuclein (SNCA) after ambroxol treatment (bars marked ‘+’). (**C**) Representative western blots demonstrating increased LAMP1 and glucosylceramidase (GCase) after ambroxol treatment and decreased HA-tagged alpha-synuclein. ‘ambroxol’ = cells treated with ambroxol.

Given the increase in glucosylceramidase activity after ambroxol treatment, we hypothesized that this drug would reduce alpha-synuclein levels in a cell line expressing this protein. After 5 days of ambroxol hydrochloride treatment (60 µM) levels of HA-tagged alpha-synuclein in the over expressing neuroblastoma cell line decreased significantly [median fall 15% (IQR 15–25%), Mann-Whitney U-test *P = *0.034] (Fig. 9B and C). This was accompanied by an increase in glucosylceramidase [median 30% increase (IQR 15–40%), *P = *0.011] and LAMP1 protein levels [median 40% increase (IQR 25–55%), *P = *0.031) (Fig. 9B and C).

## Discussion

Our results show that a reduction of glucosylceramidase protein levels and activity in fibroblasts from Gaucher disease, Parkinson’s disease with *GBA* mutations and non-manifesting carriers is because of a combination of decreases in *GBA* transcript levels and endoplasmic reticulum retention of glucosylceramidase protein. Fibroblasts derived from both Parkinson’s disease patients with *GBA* mutations and non-manifesting carriers showed similar changes in glucosylceramidase protein and activity levels, and cellular biochemical changes. However, we had only a small number of non-manifesting carrier fibroblast lines (from individuals aged 60 and 82) and cannot exclude the possibility that there may be subtle differences in glucosylceramidase metabolism and cellular biochemistry between Parkinson’s disease with GBA mutations and non-manifesting carrier cell lines, which would only be apparent in a larger cohort. Moreover, it is important to note that the development of Parkinson’s disease in *GBA* mutation carriers is age related (McNeill *et al.*, 2012). Thus the differentiation of heterozygous *GBA* mutation cell lines into Parkinson’s disease and non-manifesting carrier cell lines is artificial. As we cannot exclude that the non-manifesting carriers may eventually develop Parkinson’s disease.

Our quantitative PCR study demonstrates that the N370S and E326K mutations do not reduce transcript levels, whereas the recombinant alleles (RecNcil), frameshift (203insC, c1263del55) and L444P mutations are associated with reduced *GBA* messenger RNA levels. Previous studies had demonstrated normal levels of L444P and N370S messenger RNA, with reductions of A120G and P415R mutation bearing messenger RNA in transfected NIH 3T3 cells (Ohashi *et al.*, 1990). *GBA* null mutations and the g.5255del T mutation (predicted to cause a frameshift and stop codon) are associated with severe reductions of *GBA* transcript levels (Tayebi *et al.*, 1998; Germain *et al.*, 2001). The fact that we detected reductions in *GBA* messenger RNA levels in cells bearing the L444P mutation probably reflects the greater sensitivity of the quantitative PCR technique we used, compared with the northern blot techniques used in earlier studies. Recombinant and frame shift mutations can cause reduced messenger RNA stability through nonsense-mediated decay. Why transcript levels are reduced by the L444P missense mutation is unclear. However, missense mutations in other genes (e.g. dopamine receptor, hepatocyte nuclear factor 1-alpha) have been shown to be associated with reduced transcript levels, possibly by altering messenger RNA folding (Harries *et al.*, 2004).

Our results also provide a framework for understanding how *GBA* mutations predispose to neurodegeneration. We recently described loss of glucosylceramidase protein and enzyme activity in the substantia nigra of cases with Parkinson’s disease with and without *GBA* mutations (Gegg *et al.*, 2012). Several cell biology studies demonstrate that alpha-synuclein accumulation depletes glucosylceramidase (Mazzulli *et al.*, 2011). Thus, the reduction in glucosylceramidase activity and protein in heterozygous *GBA* mutation carriers may predispose to loss of neuronal glucosylceramidase activity secondary to age related alpha-synuclein accumulation. Once this reduction in glucosylceramidase activity crosses a hypothetical critical threshold it will result in a ‘feed-forward’ mechanism as lysosomal dysfunction leads to alpha-synuclein accumulation, which in turn exacerbates loss of glucosylceramidase. As all *GBA* mutations are predicted (or proven) to cause endoplasmic reticulum retention or reduced *GBA* transcription/messenger RNA stability (Hruska *et al.*, 2008) this hypothesis explains how a wide spectrum of *GBA* gene mutations can predispose to Parkinson’s disease. The E326K variant has been associated with increased Parkinson’s disease risk but no cases with Gaucher disease with a homozygous E326K genotype have been reported (Duran *et al.*, 2013). Here we show that the E326K variant significantly reduces glucosylceramidase activity, although to a lesser extent than other *GBA* mutations. This fits with the hypothesis that E326K reduces glucosylceramidase activity sufficiently to induce alpha-synuclein, but not glucosylceramide, accumulation, thus explaining its association with Parkinson’s disease but not Gaucher disease (Duran *et al.*, 2013). In addition, our results demonstrate that *GBA* mutations are associated with evidence of endoplasmic reticulum and oxidative stress in fibroblasts. Endoplasmic reticulum and oxidative stress are well recognized as key pathological processes in both lysosomal storage disorders and Parkinson’s disease. Upregulation of endoplasmic reticulum stress markers has been demonstrated in fibroblasts from patients with GM1-gangliosidosis, neuronal ceroid lipofuscinosis and type II Gaucher disease (Wei *et al.*, 2008). Here we confirm that endoplasmic reticulum stress markers are upregulated in type I Gaucher disease and also in Parkinson’s disease with *GBA* mutations, though to a lesser degree. This is probably secondary to endoplasmic reticulum retention of misfolded glucosylceramidase. The rate of dihydroethidium oxidation was significantly elevated in Gaucher disease, Parkinson’s disease with *GBA* mutations, the homozygous E326K and non-manifesting carrier fibroblasts accompanied by depletion of glutathione levels. Similar results were reported by Deganuto *et al.* (2007) in fibroblasts from patients with type II Gaucher disease. As we detected evidence of endoplasmic reticulum and oxidative stress in fibroblasts from non-manifesting carriers it is likely that these processes contribute to the early stages of neurodegeneration in *GBA* mutation carriers.

Reductions in fibroblast glucosylceramidase activity are associated with subtle alterations in lysosomal biochemistry. There was no alteration in lysosomal mass in Gaucher disease, Parkinson’s disease with *GBA* mutation or non-manifesting carrier fibroblasts. This is in keeping with studies of Gaucher disease bone marrow mesenchymal cells, spleen, liver and fibroblasts (Ho *et al.*, 1972; Campeau *et al.*, 2009). Nor was there evidence of impaired macroautophagy, with no significant differences in p62 or LC3 levels. This is in keeping with studies demonstrating normal autophagy in type I Gaucher disease fibroblasts (Tatti *et al.*, 2012). However, it must be acknowledged that we performed point measurements of macroautophagy and did not assess autophagic flux. Thus we cannot exclude a subtle alteration in rate of autophagy. Levels of Cathepsin D protein and total-beta hexosaminidase activity were significantly higher in Gaucher disease and Parkinson’s disease with *GBA* mutations than controls. Cathepsin D can proteolytically activate saposin c, and so theoretically ehance glucosylceramidase activity (Vitner *et al.*, 2010). Upregulation of this protein may be a homeostatic response to glucosylceramidase loss. Upregulation of cathepsin D messenger RNA has been described in the brain of Gaucher disease murine models (Vitner *et al.*, 2010), whereas in blood from patients with Gaucher disease the activity of various cathepsins is elevated (Moran *et al.*, 2000). Increases in beta-hexosaminidase activity have been reported in fibroblasts from patients with Gaucher disease (Moffitt *et al.*, 1978; Aureli *et al.*, 2012). It has been suggested that beta-hexosaminidase can catabolize other substrates accumulating in Gaucher disease. These alterations in lysosomal biochemistry are probably compensatory responses for the loss of glucosylceramidase activity, and could in theory impinge upon pathways which degrade alpha-synuclein.

The finding that Parkinson’s disease with *GBA* mutations is associated with reductions in glucosylceramidase activity in substantia nigra suggests that augmenting glucosylceramidase activity may be a valid therapeutic approach to reverse the biochemical consequences of the *GBA* mutation. Ambroxol hydrochloride is an expectorant that was identified as a potential chaperone for glucosylceramidase when it was found to protect the enzyme from thermal denaturation (Maegawa *et al.*, 2009). Maegawa *et al.* (2009) and others, have shown that ambroxol improves trafficking of glucosylceramidase to the lysosome and increases glucosylceramidase activity in Gaucher disease fibroblasts (Maegawa *et al.*, 2009; Bendikov-Bar *et al.*, 2013). We confirm this and demonstrate that ambroxol also improves glucosylceramidase activity and protein levels in Parkinson’s disease with *GBA* mutation fibroblasts, as well as in non-manifesting carrier and control fibroblasts. Though initially characterized as a pharmacological chaperone (Maegawa *et al.*, 2009), our data suggest that ambroxol can enhance glucosylceramidase activity by activating the CLEAR network through the action of TFEB. TFEB is a transcription factor that controls at least 471 genes and is the master regulator of lysosomal biogenesis (Palmieri *et al.*, 2011). Ambroxol treatment led to a significant upregulation of TFEB messenger RNA levels in all treated cells. TFEB has recently been shown to strongly induce its own expression; therefore upregulation of TFEB messenger RNA levels by ambroxol provides evidence that TFEB is being activated (Settembre *et al.*, 2013). Gene expression profiling in ambroxol treated control cells demonstrated strong upregulation of lysosomal and autophagy genes. This provides evidence of activation of the CLEAR network in association with upregulation of TFEB transcript levels. Thus ambroxol hydrochloride increases glucosylceramidase activity in Gaucher disease and Parkinson’s disease with *GBA* mutation fibroblasts by increasing expression of TFEB, which is associated with activation of components of the CLEAR network (summarized in Supplementary Fig. 2). This was accompanied by evidence of increased lysosomal enzyme protein and activity in treated cells.

Despite a significant increase in glucosylceramidase activity after ambroxol treatment, the activity in Gaucher disease fibroblasts remained only a small fraction of that of control cells. However, the reduction in cathepsin D and total beta-hexosaminidase activity after ambroxol treatment suggests improvement of lysosomal function. Ambroxol has been used to treat patients with type I Gaucher disease in a pilot trial, supporting the physiological relevance of its effect on glucosylceramidase activity (Zimran *et al.*, 2012). Treatment with ambroxol also reduced the rate of dihydroethidium oxidation in Gaucher disease, Parkinson’s disease with *GBA* mutation and control fibroblasts. Ambroxol is known to be an anti-oxidant (Stetinova *et al.*, 2004), explaining the reduction in dihydroethidium oxidation rate associated with its use. Of great importance is the demonstration that ambroxol decreases alpha-synuclein levels in an overexpressing cell line. Lysosomes are a major site of alpha-synuclein degradation and it is possible increased lysosomal mass results in an increased basal alpha-synuclein degradation rate (Alvarez-Erviti *et al.*, 2010). Thus ambroxol hydrochloride, which can cross the blood–brain barrier (Luan *et al.*, 2013), can potentially target multiple processes involved in dopaminergic neurodegeneration by improving lysosomal function in glucosylceramidase deficient cells, by acting as an anti-oxidant and increasing clearance of alpha-synuclein.

In summary, we demonstrate that *GBA* mutations predispose to cellular glucosylceramidase deficiency by inducing endoplasmic reticulum trapping of glucosylceramidase or reducing transcript levels. Glucosylceramidase deficiency is associated with alterations in lysosomal function and endoplasmic reticulum and oxidative stress. Given that heterozygous mutations in the Niemann-Pick disease gene *SMPD1* have recently been associated with Parkinson’s disease (Gan-Or *et al.*, 2013), it is possible that similar mechanisms explain how mutations in other autophagy-lysosome genes increase Parkinson’s disease risk (Shachar *et al.*, 2011). Ambroxol hydrochloride increases glucosylceramidase activity in control, Gaucher disease and Parkinson’s disease with *GBA* mutation cell lines, by activating components of the CLEAR network through TFEB combined with chaperone activity (Supplementary Fig. 2). The ability of ambroxol to increase glucosylceramidase in control cells and to reduce alpha-synuclein levels in a cell model highlights the potential benefits of manipulating the glucosylceramidase pathway to lower synuclein levels in synuclein related diseases (Schapira and Gegg, 2013).

## Supplementary Material

Supplementary Data

## Abbreviation

CLEAR: coordinated lysosomal expression and regulation

## References

[awu020-B1] Alvarez-Erviti L, Rodriguez-Oroz MC, Cooper JM, Caballero C, Ferrer I, Obeso JA (2010). Chaperone-mediated autophagy markers in Parkinson disease brains. Arch Neurol.

[awu020-B2] Aureli M, Bassi R, Loberto M, Regis S, Prinetti A, Chigorno V (2012). Cell surface associated glycohydrolases in normal and Gaucher disease fibroblasts. J Inherit Metab Dis.

[awu020-B3] Balwani M, Fuerstma MA, Kornreich R, Edelmann L, Desnick RJ (2010). Type I Gaucher disease. Arch Intern Med.

[awu020-B4] Bendikov-Bar I, Maor G, Filocamo M, Horowitz M (2013). Ambroxol acts as a pharmacological chaperone for mutant glucocerebrosidase. Blood Cells Mol Dis.

[awu020-B5] Campeau PM, Rafei M, Boivin MN, Sun Y, Grabowski GA, Galipeau J (2009). Characterization of Gaucher disease bone marrow mesenchymal stromal cells reveals an altered inflammatory secretome. Blood.

[awu020-B6] Deganuto M, Pittis MG, Pines A, Dominissini S, Kelley MR, Garcia R (2007). Altered intracellular redox status in Gaucher disease fibroblasts and impairment of adaptive response against oxidative stress. J Cell Physiol.

[awu020-B7] Dehay B, Martinez-Vicente M, Caldwell GA, Caldwell KA, Yue Z, Cookson MR (2013). Lysosomal impairment in Parkinson’s disease. Mov Disord.

[awu020-B8] Duran R, Mencacci NE, Angeli AV, Shoai M, Deas E, Houlden H (2013). The glucocerebrosidase E326K variant predisposes to Parkinson’s disease but does not cause Gaucher’s disease. Mov Disord.

[awu020-B9] Ebrahimi-Fakhari D, Wahlster L, McLean PJ (2012). Protein degradation pathways in Parkinson’s disease: curse or blessing. Acta Neuropathol.

[awu020-B10] Gan-Or Z, Ozelius LJ, Bar-Shira A, Saunders-Pullman R, Mirelman A, Kornreich R (2013). The p.L302P mutation in the lysosomal enzyme gene SMPD1 is a risk factor for Parkinson disease. Neurology.

[awu020-B11] Gegg ME, Burke D, Heales SJ, Cooper JM, Hardy J, Wood NW (2012). Glucocerebrosidase deficiency in substantia nigra of Parkinson disease brains. Ann Neurol.

[awu020-B12] Germain DP, Kaneski CR, Brady RO (2001). Mutation analysis of the beta-glucosidase gene in a patient with type 3 Gaucher disease and neutralizing antibody to alglucerase. Mut Res.

[awu020-B13] Goker-Alpan O, Stubblefield BK, Giasson BI, Sidransky E (2010). Glucocerebrosidase is present in a-synuclein inclusions in Lewy body disorders. Acta Neuropathol.

[awu020-B14] Harries LW, Hattersley AT, Ellard S (2004). Messenger RNA transcripts of the hepatocyte nuclear factor 1-alpha gene containing premature termination codons are subject to nonsense mediated decay. Diabetes.

[awu020-B15] Ho MW, Seck J, Schmidt D, Veath ML, Johnson W, Brady RO (1972). Adult Gaucher’s disease: kindred studies and demonstration of a deficiency of acid beta-glucosidase in cultured fibroblasts. Am J Hum Genet.

[awu020-B16] Houlden H, Singleton AB (2012). The genetics and neuropathology of Parkinson’s disease. Acta Neuropathol.

[awu020-B17] Hruska K, LaMarca M, Scott RC, Sidranksy E (2008). Gaucher disease: mutation and polymorphism spectrum in the glucocerebrosidase gene (GBA). Hum Mut.

[awu020-B18] Kurzawa-Akanbi M, Hanson PS, Blain PG, Lett DJ, McKeith IG, Chinnery PF (2012). Glucocerebrosidase mutations alter the endoplasmic reticulum and lysosomes in Lewy body disease. J Neurochem.

[awu020-B19] Lee MJ, Lee JH, Rubinsztein DC (2013). Tau degradation: the ubiquitin-proteasome system versus the autophagy lysosome system. Prog Neurobiol.

[awu020-B20] Luan Z, Li L, Higaki K, Nanba E, Suzuki Y, Ohno K (2013). The chaperone activity and toxicity of ambroxol on Gaucher cells and normal mice. Brain Dev.

[awu020-B21] Maegawa GH, Tropak MD, Buttner JD, Rigat BA, Fuller M, Pandit D (2009). Identification and characterization of ambroxol as an enzyme enhancement agent for Gaucher disease. J Biol Chem.

[awu020-B22] Mazzulli JR, Xu YH, Sun Y, Knight AL, McLean PJ, Caldwell GA (2011). Gaucher disease glucocerebrosidase and a-synuclein form a bidirectional pathogenic loop in synucleinopathies. Cell.

[awu020-B23] McNeill A, Duran R, Hughes DA, Mehta A, Schapira AH (2012). A clinical and family history study of Parkinson’s disease in glucocerebrosidase mutation carriers. J Neurol Neurosurg Psych.

[awu020-B24] Moffitt KD, Chambers JP, Diven WF, Glew RH, Wenger DA, Farrell DF (1978). Characterization of lysosomal hydrolases that are elevated in Gaucher's disease. Arch Biochem Biophys.

[awu020-B25] Moran MT, Schofield JP, Hayman AR, Shi GP, Young E, Cox TM (2000). Pathologic gene expression in Gaucher disease: upregulation of cysteine proteinases including osteoclastic cathepsin K. Blood.

[awu020-B26] Nalls MA, Duran R, Lopez G, Kurzawa-Akanbi M, McKeith IG, Chinnery PF (2013). A multicentre study of glucocerebrosidase mutations in dementia with Lewy bodies. JAMA Neurol.

[awu020-B27] Ohashi T, Hong CM, Weiler S, Tomich JM, Aerts JM, Tager JM (1990). Characterization of human glucocerebrosidase from different mutant alleles. J Biol Chem.

[awu020-B28] Otomo A, Pan L, Hadano S (2012). Dysregulation of the autophagy-endolysosomal system in amyotrophic lateral sclerosis and related motor neuron diseases. Neurol Res Int.

[awu020-B29] Palmieri M, Impey S, Kang H, di Ronza A, Pelz C, Sardiello M (2011). Characterisation of the CLEAR network reveals an integrated control of cellular clearance pathways. Hum Mol Genet.

[awu020-B30] Ron I, Horowitz M (2005). ER retention and degradation as the molecular basis underlying Gaucher disease heterogeneity. Hum Mol Genet.

[awu020-B31] Rosenbloom B, Balwani M, Bronstein JM, Kolodny E, Sathe S, Gwosdow AR (2011). The incidence of parkinsonism in patients with Type I Gaucher disease: data from the IGCC Gaucher registry. Blood Cell Mol Dis.

[awu020-B32] Sawkar AR, Schmitz M, Zimmer KP, Reczek D, Edmunds T, Balch WE (2006). Chemical chaperones and permissive temperatures alter localisation of Gaucher disease associated glucocerebrosidase variants. ACS Chem Biol.

[awu020-B33] Shachar T, Lo Bianco C, Recchia A, Wiessner C, Raas-Rothschild A, Futerman AH (2011). Lysosomal storage disorders and Parkinson’s disease: Gaucher disease and beyond. Mov Disord.

[awu020-B34] Schapira AH, Gegg ME (2013). Glucocerebrosidase in the pathogenesis and treatment of Parkinson disease. Proc Natl Acad Sci USA.

[awu020-B35] Settembre C, De Cegli R, Mansueto G, Saha PK, Vetrini F, Visvikis O (2013). TFEB controls cellular lipid metabolism through a starvation-induced autoregulatory loop. Nat Cell Biol.

[awu020-B36] Sidransky E, Lopez G (2012). The link between the GBA gene and parkinsonism. Lancet Neurol.

[awu020-B37] Sidransky E, Nalls MA, Aasly JO, Aharon-Peretz J, Annesi G, Barbosa ER (2009). Multicenter analysis of glucocerebrosidase mutations in Parkinson’s disease. N Engl J Med.

[awu020-B38] Song W, Wang F, Savini M, Ake A, Savini M, di Ronza A (2013). TFEB regulates lysosomal proteostasis. Hum Mol Genet.

[awu020-B39] Stetinova V, Herout V, Kvetina J (2004). In vitro and in vivo antioxidant activity of ambroxol. Clin Exp Med.

[awu020-B40] Subramanian A, Tamayo P, Mootha VK, Mukherjee S, Ebert BL, Gillette MA (2005). Gene set enrichment analysis: a knowledge-based approach for interpreting genome-wide expression profiles. Proc Nat Acad Sci USA.

[awu020-B41] Tajima A, Yokoi T, Ariga M, Ito T, Kaneshiro E, Eto Y (2009). Clinical and genetic study of Japanese patient with Type 3 Gaucher Disease. Mol Genet Metab.

[awu020-B42] Tatti M, Motta M, Di Bartolomeo S, Scarpa S, Cianfanelli V, Cecconi F (2012). Reduced cathepsins B and D cause impaired autophagic degradation that can be almost completely restored by overexpression of these two proteases in sap-C deficient fibroblasts. Hum Mol Genet.

[awu020-B43] Tayebi N, Reissner KJ, Lau KL, Stubblefield BK, Klineburgess AC, Martin BM (1998). Genotypic heterogeneity and phenotypic variation among patients with Type 2 Gaucher disease. Ped Res.

[awu020-B44] Tsuang D, Leverenz JB, Lopez OL, Hamilton RL, Bennett DA, Schneider JA (2012). GBA mutations increase risk for Lewy body disease with and without Alzheimer disease pathology. Neurology.

[awu020-B45] Vitner EB, Dekel H, ZiGaucher diseaseon H, Shachar T, Farfel-Becker T, Eilam R (2010). Altered expression and distribution of cathepsins in neuronopathic forms of Gaucher disease. Hum Mol Genet.

[awu020-B46] Wei H, Kim SJ, Zhang Z, Tsai PC, Wisniewski KE, Mukherjee AB (2008). ER and oxidative stresses are common mediators of apoptosis in both neurodegenerative and non-neurodegenerative lysosomal storage disorders and are alleviated by chemical chaperones. Hum Mol Genet.

[awu020-B47] Zimran A, Altarescu G, Elstein D (2013). Pilot study using ambroxol as a pharmacological chaperone in type I Gaucher disease. Blood Cells Mol Dis.

