# Supplementary material for: Ambroxol improves lysosomal biochemistry in glucocerebrosidase mutation-linked Parkinson disease cells
Source: Brain. 2014 Feb 25;137(5):1481–95. doi: 10.1093/brain/awu020 (PMC3999713; doi:10.1093/brain/awu020)
Supplement: Supplementary Data [file supp_137_5_1481__index.html]

Ambroxol improves lysosomal biochemistry in glucocerebrosidase mutation-linked Parkinson disease cells — Supplementary Data 

# Ambroxol improves lysosomal biochemistry in glucocerebrosidase mutation-linked Parkinson disease cells

## Supplementary Data

files

**Files in this Data Supplement:**

- Supplementary Data - doc file
