# Supplementary material for: Ambroxol improves lysosomal biochemistry in glucocerebrosidase mutation-linked Parkinson disease cells
Source: Brain. 2014 Feb 25;137(5):1481–95. doi: 10.1093/brain/awu020 (PMC3999713; doi:10.1093/brain/awu020)
Supplement: Supplementary Data [file supp_awu020_brain-2013-01720-File002.doc]

**Supplementary material for online publication.**

|  | **Table S1. Primers used for qPCR.** |  |
| --- | --- | --- |
| **Target** | **Sequence/taqman primer identification code** | **Annealing Temp (°C)** |
| β-actin | 5’-TCT ACA ATG AGC TGC GTG TG-3’  5’-GGT GAG GAT CTT CAT GAG GT-3’ | 58 |
| Glucosylceramidase | 5’-TGC TGC TCT CAA CAT CCT TGC C-3’  5’-TAG GTG CGG ATG GAG AAG TCA A-3’ | 58 |
| NAD(P)H dehydrogenase (quinone 1)(NQo1) | 5’-CAGTCACCGAGAGCCTAGT-3’  5’-GAGTGAGCCAGTACGATCAGTG-3’ | 62 |
| Transcription factor EB | Hs00292981_m1. | 60 |
| SCARB/LIMP2 | Hs01072100_m1 | 60 |
| Aspartylglucosamindase | Hs00240537_m1 | 60 |
| Cathepsin K | Hs00166156_m1 | 60 |
| Hexosaminidase A | Hs00166843_m1 | 60 |
| Iduronate sulfatase-2 | Hs00164940_m1 | 60 |
| Beta-actin | Hs01060665_g1 | 60 |

|  | **Table S2. Lysosomal genes upregulated by ambroxol. GeneChip Human Genome U133 Plus 2.0 Array.** |  |  |
| --- | --- | --- | --- |
| **Gene symbol** | **Gene name** | **Fold change** | **p-value** |
| CTSK | Cathepsin K | 5.4 | 2.1x10^-7^ |
| NEU1 | Sialidase 1 | 3.2 | 0.0007 |
| GM2A | Gangliosidase activator 2A | 3.2 | 0.00058 |
| ASAH1 | N-acylsphingosine amidohydrolase-1 | 3.17 | 0.00012 |
| IDS | Iduronate-2 sulfatase | 2.5 | 0.0047 |
| NPC1 | Niemann-Pick disease, Type C1 | 2.49 | 0.001 |
| IGF2R | Insulin-like growth factor 2 receptor | 2.48 | 0.0012 |
| CTSL1 | Cathepsin L1 | 2.44 | 0.001 |
| SLC17A5 | Solute carrier family 17, member 5 | 2.44 | 0.008 |
| NPC2 | Niemann-Pick disease, Type C2 | 2.41 | 0.0005 |
| CTSA | Cathepsin A | 2.35 | 0.00017 |
| TPP1 | Tripeptidyl peptidase 1 | 2.34 | 0.00011 |
| PSAP | Prosaposin | 2.34 | 0.0001 |
| CTSD | Cathepsin D | 2.29 | 0.002 |
| CTSF | Cathepsin F | 2.26 | 0.005 |
| GBA1 | Glucosylceramidase | 2.21 | 0.005 |
| GNPTAB | N-acetylglucosamine-1-phosphate transferase | 2.1 | 0.003 |
| PLA2G15 | Phospholipase A2, group 15 | 2.06 | 0.0024 |
| DNASEII | Deoxyribonuclease II, lysosomal | 2.03 | 0.002 |
| CTSB | Cathepsin B | 2.01 | 0.00089 |
| FUCA1 | Fucosidase, alpha-1 | 2.0 | 0.006 |
| CTSO | Cathepsin O | 1.99 | 0.02 |
| LAMP1 | Lysosomal associated membrane protein 1 | 1.99 | 0.02 |
| HEXA | Hexosaminidase A (alpha polypeptide) | 1.99 | 0.02 |
| LIPA | Lipase A, lysosomal | 1.88 | 0.0005 |
| AGA | Aspartylglucosaminidase | 1.88 | 0.003 |
| MANBA | Mannosidase, beta A, lysosomal | 1.86 | 0.001 |
| IDUA | Iduronidase, Alpha-L | 1.8 | 0.01 |
| M6PR | Mannose-6-phosphate receptor | 1.8 | 0.019 |
| HEXB | Hexosaminidase B (beta polypeptide) | 1.8 | 0.0009 |
| GNS | Glucosamine-6-sulfatase | 1.78 | 0.0019 |
| GALC | Galactosylceramidase | 1.76 | 0.0004 |
| LAMP2 | Lysosomal-associated membrane protein 2 | 1.71 | 0.004 |
| SCARB2 | Scavenger receptor class B, member 2 | 1.62 | 0.0077 |
| GLB1 | Galactosidase, beta 1 | 1.59 | 0.00032 |
| CLN5 | Ceroid lipofuscinosis, neuronal 5 | 1.55 | 0.016 |
| ATP6VOD1 | ATPase, H+ transporting, lysosomal 38kDa, VO subunit 1. | 1.54 | 0.031 |
| SUMF1 | Sulfatase modifying factor 1 | 1.54 | 0.01 |
| ANKHD1 | Ankyrin repeat and KH domain-containing protein 1 | 1.54 | 0.002 |
| CD164 | CD164 molecule | 1.53 | 0.0002 |
| GALNS | Galactosamine | 1.51 | 0.0012 |
| NAGA | N-Acetylgalactosaminidase, Alpha | 1.44 | 0.034 |
| CTNS | Cystinosin, lysosomal | 1.4 | 0.031 |
| CLN3 | Ceroid lipofuscinosis, neuronal 3 | 1.36 | 0.009 |
| GGA2 | golgi-associated, gamma adaptin ear containing, ARF binding protein 2 | 1.36 | 0.028 |
|  | **Table S2. Autophagy genes** |  |  |
| ULK1 | UNC-51-like Kinase 1 | 1.7 | 0.005 |
| ATG12 | Autophagy related 12 | 1.6 | 0.008 |
| GABARAPL1 | GABA receptor associated like protein 1 | 1.7 | 0.01 |
| PIK3C3 | Phosphoinositide -3 kinase, class 3 | 1.24 | 0.02 |
| PIK3R4 | Phosphoinositide-3 kinase, regulatory subunit 4 | 1.27 | 0.024 |
| ATG5 | Autophagy related gene 5 | 1.3 | 0.023 |
| ATG7 | Autophagy related gene 7 | 1.26 | 0.024 |
| GABARAP | GABA-receptor associated protein | 1.2 | 0.024 |
| ATG12 | Autophagy related gene 12 | 1.37 | 0.034 |
| BECN1 | Beclin 1, autophagy related | 1.2 | 0.05 |

| **Table S3. qPCR validation of genes upregulated by ambroxol.** | | | |
| --- | --- | --- | --- |
|  | Control | GD | PD-GBA |
| Hexosaminidase A | 2.5 fold increase, p=0.037 | 3.5 fold increase, p=0.03 | 2.0 fold increase, p=0.04 |
| Cathepsin K | 6.0 fold increase, p=0.025 | 3.0 fold increase, p=0.03 | 6.0 fold increase, p=0.03 |
| SCARB2/LIMP2 | 2.5 fold increase, p=0.034 | 4.0 fold increase, p=0.03 | 2.5 fold increase, p=0.035 |
| Iduronidase, Alpha-L | 1.9 fold increase p=0.037 | 8 fold increase, p=0.02 | 2.0 fold increase, p=0.04 |


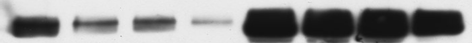


GCase


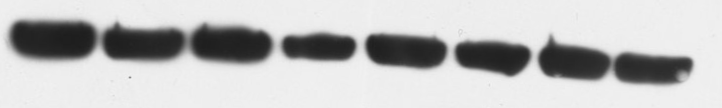


Actin

60 30 10 0 60 30 10 0

Gaucher disease

Control

**Supplementary figure 1. Titration of ambroxol dose.**

**Top panel shows a Western blot of glucosylceramidase (GCase) protein levels. A Gaucher disease line (GD03) and a control cell line were incubated with 0 uM, 10 uM, 30 uM and 60 uM of ambroxol hydrochloride for 5 days. The greatest increase in GCase protein levels was noted with 60 uM of ambroxol hydrochloride. A dose of 60 uM ambroxol hydrochloride was therefore chosen for rescue experiments.**

+

+

Decrease in SNCA in overexpressing line


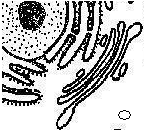

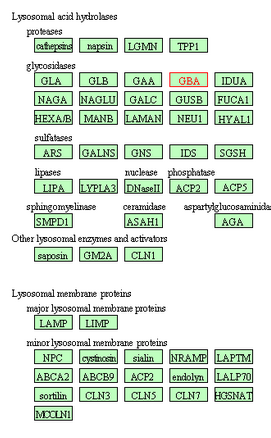


Activation of CLEAR network gene transcription via TFEB upregulation


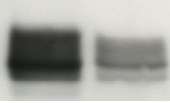


Decrease in endoplasmic reticulum retained fraction of GCase by chaperone activity of ambroxol (lane marked +)

Increased GCase activity and lysosomal mass in treated cells


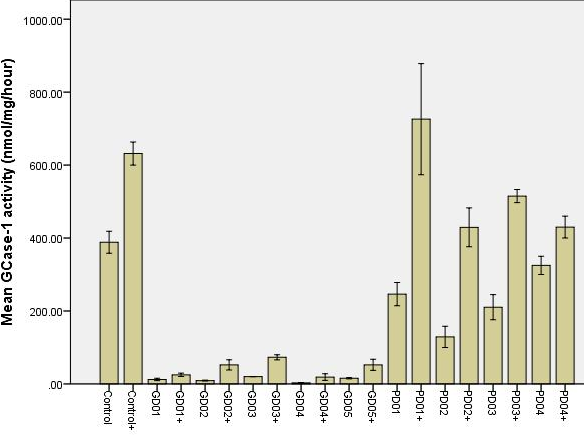

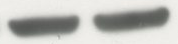


SNCA

Actin

NUCLEUS

ER-Golgi body

LYSOSOME

**Supplementary figure 2. Summary of cellular effects of ambroxol hydrochloride.**

**Ambroxol activates the CLEAR network by upregulating TFEB, this leads to increased expression of lysosomal enzymes, membrane proteins and trafficking proteins (summary list in green above diagram). Ambroxol also acts post-transcriptionally as a chaperone, shown by reduced endoplasmic reticulum retained fraction of glucosylceramidase (GCase) (Western blot to bottom left of diagram). Activation of the CLEAR network by ambroxol results in increased lysosomal mass and lysosomal enzyme activity, including GCase. This was associated with reduced alpha-synuclein levels in an alpha-synuclein overexpressing cell line. “+” = treated with ambroxol hydrochloride.**
